# Heterochiasmy and Sexual Dimorphism: The Case of the Barn Swallow (*Hirundo rustica*, Hirundinidae, Aves)

**DOI:** 10.3390/genes11101119

**Published:** 2020-09-24

**Authors:** Lyubov P. Malinovskaya, Katerina Tishakova, Elena P. Shnaider, Pavel M. Borodin, Anna A. Torgasheva

**Affiliations:** 1 Institute of Cytology and Genetics, Siberian Branch of Russian Academy of Sciences, 630090 Novosibirsk, Russia; l.malinovskaia@g.nsu.ru (L.P.M.); k.tishakova@g.nsu.ru (K.T.); borodin@bionet.nsc.ru (P.M.B.); 2 Department of Cytology and Genetics, Novosibirsk State University, 630090 Novosibirsk, Russia; 3 SibEcoCenter LLC, 630090 Novosibirsk, Russia; equ001@gmail.com

**Keywords:** heterochiasmy, sexual selection, barn swallow, sand martin, pale martin, recombination, crossing over, MLH1, SYCP3, bird genome evolution

## Abstract

Heterochiasmy, a sex-based difference in recombination rate, has been detected in many species of animals and plants. Several hypotheses about evolutionary causes of heterochiasmy were proposed. However, there is a shortage of empirical data. In this paper, we compared recombination related traits in females and males of the barn swallow *Hirundo rustica* (Linnaeus, 1758), the species under strong sexual selection, with those in the pale martin *Riparia diluta* (Sharpe and Wyatt, 1893)*,* a related and ecologically similar species with the same karyotype (2N = 78), but without obvious sexual dimorphism. Recombination traits were examined in pachytene chromosome spreads prepared from spermatocytes and oocytes. Synaptonemal complexes and mature recombination nodules were visualized with antibodies to SYCP3 and MLH1 proteins, correspondingly. Recombination rate was significantly higher (*p* = 0.0001) in barn swallow females (55.6 ± 6.3 recombination nodules per autosomal genome), caused by the higher number of nodules at the macrochromosomes, than in males (49.0 ± 4.5). They also showed more even distribution of recombination nodules along the macrochromosomes. At the same time, in the pale martin, sexual differences in recombination rate and distributions were rather small. We speculate that an elevated recombination rate in the female barn swallows might have evolved as a compensatory reaction to runaway sexual selection in males.

## 1. Introduction

Recombination plays an important role in reshuffling alleles from generation to generation. Genetic variation of the number and distribution of recombination events has been detected in many species of plants and animals [1,2,3].

Sex-based differences in recombination (heterochiasmy) have attracted special attention since the early days of genetics. Heterochiasmy is distributed erratically among taxa and varies in magnitude and direction [3,4]. A higher recombination rate is more often observed in females than in males [4,5,6]. Sex-based differences in the distribution of the recombination event along the chromosomes have also been detected. Males usually show more polarized distribution with stronger peaks at telomeres. Females display more even distribution slightly shifted towards centromeres [7,8].

The proximate and ultimate causes of heterochiasmy are not clear. The higher rate and flatter distribution of recombination in females have been ascribed to longer synaptonemal complexes (SC) and weaker chiasma interference [7,9].

Several hypotheses about evolutionary causes of heterochiasmy were proposed. It has been considered as a collateral result of selection against recombination between the sex chromosomes in the heterogametic sex [10]. However, many examples of higher recombination rate in the heterogametic females contradict this generalization [4,8].

Lenormand [11] suggested that sex differences in recombination can result from selection on the haploid phase of the life cycle. This mechanism might operate in plants, but it is hardly applicable to animals [8,12]. Selection against meiotic drive in female meiosis can also contribute to the increase of recombination rate and preferential location of crossovers near centromeres [6,8,13].

Trivers [14] suggested that sexual selection should favor tighter linkage between loci important for male reproductive success. Lenormand [11] carried out a population genetic model analysis of this hypothesis. He concluded that sexual selection might result in decreased recombination in males if the sex difference in the strength of epistasis between alleles of different loci depended on the phase of their linkage (coupling or repulsion). Sardell and Kirkpatrick [8] modified this model, suggesting that sex differences in epistasis between coding regions and their cis regulatory regions.

The abundance of theoretical models of heterochiasmy contrasts with scarcity of data. Genetic linkage studies on non-model species are expensive and time consuming. Cytological analysis of recombination nodules makes it possible to analyze large number of meiosis in both sexes [15,16].

In this study, using immunolocalization of the proteins involved in SC and recombination nodules at the pachytene chromosomes, we compared recombination rate and distribution in females and males of the barn swallow *Hirundo rustica* (Linnaeus, 1758), the species under strong sexual selection [17,18], with those in the pale martin *Riparia diluta* (Sharpe and Wyatt, 1893), the related and ecologically similar species showing no sexual dimorphism in morphology [19,20].

## 2. Materials and Methods

### 2.1. Specimens

Adult males were captured by bird nets near the nests, at the beginning of breeding season at the end of May. Nestling females on days 3–6 after hatching were collected from the nests. The number of specimens examined and the coordinates of the trapping localities are listed in Supplementary Table S1.

The barn swallows were identified morphologically. The pale martins were identified by DNA barcoding. DNA was isolated from heart and kidney samples by routine phenol-chloroform technique. Primers and PCR conditions for the amplification of a fragment of the mitochondrial COI gene were used according to Hebert et al. [21]. The amplicons were Sanger sequenced. The sequences were processed using MEGA7 (https://megasoftware.net) and then analyzed using the distance-based and tree-based identification tools of the BOLD v.4 database (http://boldsystems.org) [22]. The DNA sequences confirmed correct identification of the individuals as being the pale martin (GenBank accession number MN216343) according to Pavlova et al. [19].

Capture, handling, and euthanasia of the birds followed the protocols approved by the Ethics Committee on Animal Care and Use of the Institute of Cytology and Genetics (approval No. 45/2 of 10 January, 2019). Experiments described in this manuscript were carried out in accordance with the approved national guidelines for the care and use of animals.

### 2.2. Chromosome Spreading and Staining

Pachytene chromosome spreads were prepared from spermatocytes or juvenile oocytes according to the protocol described by Peters et al. [23]. Immunostaining was performed according to Anderson et al. [24] using the following set of primary antibodies: rabbit polyclonal anti-SYCP3 (1:500; Abcam, Cambridge, UK), mouse monoclonal anti-MLH1 (1:50; Abcam, Cambridge, UK), and human anticentromere (ACA) (1:100; Antibodies Incorporated, Davis, CA, USA). For the secondary antibodies, we used Cy3-conjugated goat anti-rabbit (1:500; Jackson ImmunoResearch Laboratories, West Grove, PA, USA), FITC-conjugated goat anti-mouse (1:50; Jackson ImmunoResearch), and AMCA-conjugated donkey anti-human (1:100; Jackson ImmunoResearch). Antibodies were diluted in PBT (3% bovine serum albumin and 0.05% Tween 20 in phosphate-buffered saline). A solution of 10% PBT was used for blocking. Primary antibodies were incubated overnight at 37 °C; and secondary antibodies for 1 h at 37°C in a humid chamber. Slides were mounted in Vectashield antifade mounting medium (Vector Laboratories, Burlingame, CA, USA) to reduce fluorescence fading.

The preparations were examined with an Axioplan 2 imaging microscope (Carl Zeiss, Oberkochen, Germany) equipped with a CCD camera (CV M300, JAI Corporation, Yokohama, Japan, CHROMA filter sets, and the ISIS4 image-processing package (MetaSystems, Altlußheim, Germany). Brightness and contrast of the images were enhanced using Corel PaintShop Photo Pro X6 (Corel Corporation, Ottawa, ON, Canada).

### 2.3. Chromosome Measurements and Generation of Recombination Maps

The centromeres were identified by ACA foci. The MLH1 signals were scored if they were localized on SCs. The length of the SC of each chromosome arm was measured in micrometers, and the positions of centromeres and MLH1 foci in relation to the centromere were recorded using MicroMeasure 3.3 [25]. Individual SCs of macrochromosomes were identified by their relative lengths and centromeric indexes. To generate recombination maps of the macrochromosomes, we calculated the absolute position of each MLH1 focus by multiplying the relative position of each focus by the average absolute length of the chromosome arm. These data were pooled for each arm and graphed to represent a recombination map.

The STATISTICA 6.0 software package (StatSoft, Tulsa, OK, USA) was used for descriptive statistics. Values in the text and tables are presented as means ± S.D. Differences between the sexes and species in the average number of MLH1 foci and SC length were estimated by Mann–Whitney non-parametric test. The result *p* < 0.01 was considered to be statistically significant.

## 3. Results

### 3.1. Pachytene Karyotype of the Barn Swallow

**Figure 1:**
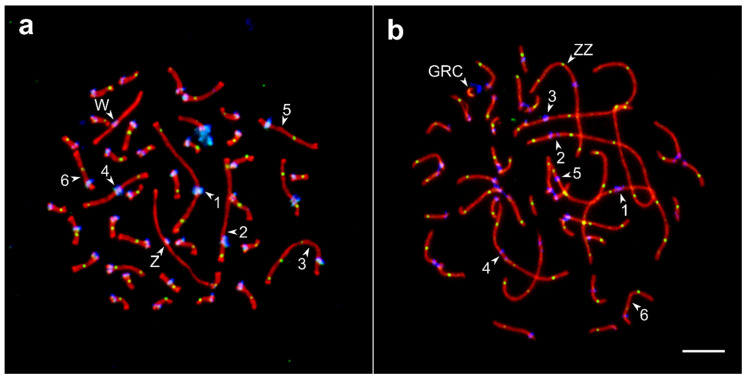
Synaptonemal complexes (SC) spreads of the oocyte (**a**) and spermatocyte (**b**) of the barn swallow after immunolocalization of the lateral elements of the SC (SYCP3—red signal), recombination nodules (MLH1—green), and centromeres (ANA-C—blue). Numbers indicate SCs of the macrochromosomes, letters—ZZ, ZW, and GRC. Bar—5 µm.

The barn swallow karyotype has not been described yet. Its pachytene karyotype contained 38 autosomal SCs and a ZZ/ZW pair (Figure 1). We identified seven largest macroSCs by their relative lengths and centromeric indices. The SC1, SC4, and SCZZ were large metacentrics; they differed from each other in length and centromeric indices (*p* < 0.001). The SC2 and SC3 were large submetacentrics and differed from each other in centromeric indices. The SC5 and SC6, medium-sized submetacentrics, also differed from each other in centromeric indices. The macroSCs 7, 9, 10, and all microSCs but two, were acrocentric, with gradually decreasing chromosomal sizes (Figure S1). Thus, barn swallow pachytene karyotype was similar to that described in pale martin and sand martin *Riparia riparia* (Linnaeus, 1758) [26], with their only difference concerning germline restricted chromosome (GRC). The GRC was one of the microchromosomes found in the barn swallow and the largest acrocentric chromosome in pale martin and sand martin [26,27]. In male barn swallows, GRC always appeared as a univalent lacking MLH1 signal and was heavily labelled by centromere antibodies (Figure 1b).

### 3.2. Recombination Rate

Table 1 shows recombination characteristics of studied females and males of barn swallow and pale martin. The barn swallow showed a pronounced sexual dimorphism in the total number of MLH1 foci at autosomes: in the oocytes, it was 13.5% higher than in spermatocytes (Mann–Whitney test z = 12.1; *p* < 0.00001). The pale martin demonstrated less pronounced (4.5%), although significant, sex difference in this trait (z = 6.9, *p* < 0.00001). In this species, spermatocytes contained more MLH1 foci at autosomes than oocytes. The pale martin males were sampled from two different locations: three from Novosibirsk and three from Tomsk (Table S1). The difference between them was not significant (z = 0.4; *p* = 0.7).

In both species, we detected significant sex differences in the total SC length: in males, it was longer than in females (z = 10.6 in barn swallow and 10.9 in pale martin; *p* < 0.00001).

The sex differences of the barn swallow in the total number of autosomal MLH1 foci was mainly due to the macrochromosomes. Almost all microchromosomes in the barn swallow and pale martin, as well as in all bird species examined, contained single recombination nodules. The microchromosomes with two MLH1 foci were rare.

In the female barn swallows, five out of six macroSCs had significantly more MLH1 foci than in the males (*p* < 0.00001). The SC 6 was an exception (z = 0.8, *p* = 0.4). None of the pale martin macroSCs showed significant sex differences in the number of MLH1 foci (*p* > 0.01) (Table 2 and Table S2).

The sex difference in the SC length in the barn swallow was also significant, but with opposite signs: the males had longer macroSCs than females. Thus, the high recombination rate of the macroSCs in the female barn swallows was achieved by a decrease of the distance between the recombination nodules, which in turn indicate a decrease in crossover interference.

Besides the autosomes, we also examined recombination characteristics of the sex chromosomes. In both species, the sex bivalent was similar to that of most songbird species studied. The SC lateral element of Z chromosome was substantially longer than that of W at zygotene at the beginning of pachytene, when they were paired by their distal ends. As the synapsis progressed, it involved equalization of the elements (contraction of Z and elongation of W chromosomes). At the mid-pachytene, ZW bivalent was completely paired (Figure 1), and usually contained a single MLH1 focus at its distal end. The ZZ bivalent usually had two (rarely three) MLH1 foci, similar to the autosomal bivalents of a comparable length (Table 2). The difference in the number of MLH1 foci in ZZ of barn swallow and pale martin was not significant (*p* = 0.02).

### 3.3. Recombination Distribution along the Macrochromosomes

**Figure 2:**
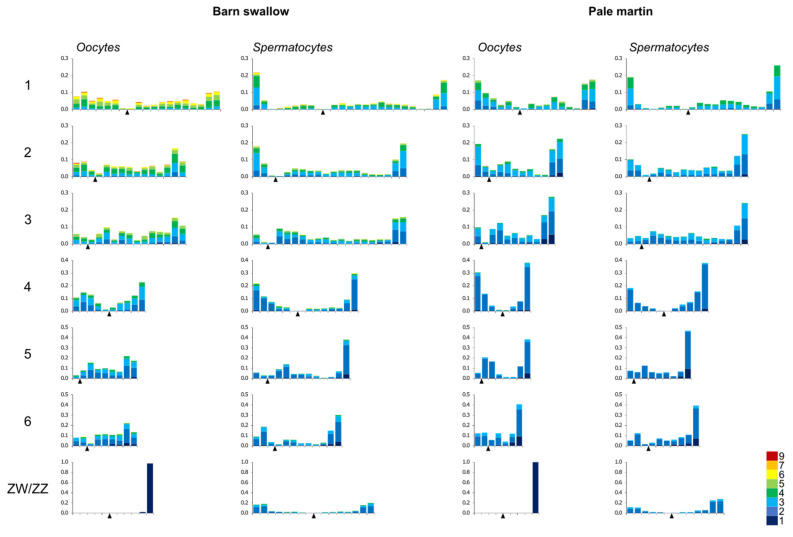
Distribution of MLH1 foci along individual SCs in pachytene oocytes and spermatocytes of barn swallow and pale martin. On the *x*-axis: the relative position of MLH1 foci at the six largest macroSCs and ZW/ZZ bivalents in relation to the centromere (black triangle). The width of the interval is approximately 1 μm. On the *y*-axis: the proportion of MLH1 focus number in each interval. Colors indicate bivalents with 1–5 MLH1 foci per bivalent. The scale shows the color codes. The numbers to the left of the *y*-axis stand for chromosome numbers.

The pattern of MLH1 foci distribution in the spermatocytes of both species, and in the oocytes of the pale martin, was rather similar. The peaks of the MLH1 foci were observed near the SC ends, while the rest of the chromosome arms contained a relatively low number of them (Figure 2).

The recombination landscape of the macrochromosomes of the barn swallow oocytes was quite different. The telomeric peaks were less pronounced and the distribution of the MLH1 foci was more even. Thus, the recombination in the macrochromosomes of the barn swallow oocytes was not only higher than spermatocytes of the same species and in the oocytes of the pale martin, but also more evenly distributed.

## 4. Discussion

We found that the barn swallow, the species with sex differences in morphology, demonstrated a substantial heterochiasmy, with sex differences in recombination rate and distribution along the macrochromosomes. Oocytes showed 13.5% higher recombination rate and more even distribution of recombination nodules along the macrochromosomes than spermatocytes. These two factors should lead to a higher outcome of crossovers and a faster break up of linkage disequilibrium in female barn swallow meiosis.

The related hirundine species, the pale martin, did not show obvious sexual dimorphism in morphology and difference in recombination characteristics.

Sexual dimorphism is usually considered as a result of sexual selection [28]. Several lines of evidence indicate that male barn swallows are under strong selection for display [17,29]. Therefore, their morphological traits might be considered as derived, and female traits as ancestral.

What about recombination characteristics? Should we consider the lower recombination rate and polarized distribution of crossovers typical to the barn swallow males as derived traits, and higher recombination rate and even distribution typical to the barn swallow females as ancestral? If the answer is yes, then our results match the prediction of the sexual selection hypothesis of heterochiasmy.

However, there are facts that cast doubts on this explanation. The barn swallow males are similar in their recombination pattern to sexually monomorphic pale martins (Table 1). Recombination rate of the barn swallow females is highest among the female songbird studied (Table 3). To save the standard explanation, we should suppose that the ancestors of the barn swallow had evolved for an increase of recombination rate in both sexes, and then sexual selection decreased it in males.

Alternatively, we may suggest that the low recombination rate is an ancestral trait, which facilitated an establishment and maintenance of linkage disequilibrium between the alleles, controlling male display and female preference to the display. Because the sex difference in recombination in the barn swallow is most pronounced in macrochromosomes, one might suppose that the genes conserved by sexual selection are located there. The strong selection for linkage disequilibrium would lead to accumulation of deleterious mutations in these regions. An increase of recombination in macrochromosomes in female meiosis might be aimed to purge them.

Of course, this hypothesis is highly speculative. We need more data on the occurrence, magnitude, and direction of heterochiasmy among the birds with and without sexual dimorphism in morphology.

Besides the two species described here, recombination rate has been studied so far in both sexes in only a few species (Table 3). Some studies used a cytological approach, while others used a linkage analysis with different density of markers. To make these data comparable, we transformed the number of recombination nodules into genetic map distances (one recombination nodule = 50 cM) and calculated heterochiasmy index as (female genetic map length – male genetic map length)/sex average genetic map length.

Table 3 shows no obvious phylogenetic clustering for magnitude and sign of the heterochiasmy index. There is no obvious correlation between the heterochiasmy and sexual dimorphism. All four species of domesticated Galloanserae show sexual dimorphism in morphology and differ from each other in heterochiasmy: it is female-biased in the domestic goose, male-biased in turkey, and negligible in Japanese quail and domestic chicken. Four of the seven songbird species studied showed female-biased heterochiasmy, one species showed male-biased and two species exhibited no heterochiasmy. In this group of species, we also did not see a coincidence between sexual dimorphism in morphology and heterochiasmy. Two species with a highly positive heterochiasmy index, the Siberian jay and great reed warbler, show no sexual dimorphism, while the obviously dimorphic zebra finch shows no heterochiasmy.

Apparently, the macro-phylogenetic comparison fails to reveal the coevolution of sexual dimorphism in morphology and recombination rate. To shed a light on the evolution of heterochiasmy in birds, we need to compare the related and ecologically similar species. This comparison must take into account not only overall recombination, but also the pattern of its distribution between and along the particular chromosomes (even vs. polarized, centromere vs. telomere biased). We also need an insight into genetic control of sex-restricted traits and its chromosomal localization. Swallows (17 species in genus *Hirundo* and seven subspecies in *H. rustica*), martins (six species in genus *Riparia* and four subspecies in *R. diluta*), and the genera between them (such as *Delichon*, for example), with their large and permanent colonies and strong monogamy, provide a good model for such studies.

## Figures and Tables

**Table 1:** Number of MLH1 foci at autosomes and total length of autosomal SC (m ± S.D.) in female and male barn swallows and pale martins.

Species	Sex	N Specimens	N Nuclei	MLH1 Foci Number	SC Length (µm)
Barn swallow	female	3	182	55.6 ± 6.3 *^§^	184.8 ± 32.3 *^§^
Barn swallow	male	5	275	49.0 ± 4.5 ^§^	215.5 ± 33.8 ^§^
Pale martin	female	3	145	46.6 ± 3.6 *^§^	169.3 ± 22.2 *^§^
Pale martin	male	6	293	48.9 ± 2.4	210.3 ± 28.9

* sex difference in the same species, Mann–Whitney test, *p* < 0.001; ^§^ species difference in the same sex, Mann–Whitney test, *p* < 0.001.

**Table 2:** Number of MLH1 foci and SC length (m ± S.D.) at the macroSCs in females and males of barn swallow and pale martin (number of SCs examined is shown in Table S1).

	MLH1 Foci Number				SC Length (µm)			
SC	Barn Swallow		Pale Martin		Barn Swallow		Pale Martin	
	Female	Male	Female	Male	Female	Male	Female	Male
1	4.4 ± 1.2 *^§^	3.4 ± 1.0 ^§^	2.9 ± 0.9	2.9 ± 0.8	18.6 ± 3.6 *^§^	25.3 ± 4.6 ^§^	15.1 ± 2.4 *	19.8 ± 5.1
2	3.6 ± 1.1 *^§^	2.9 ± 0.9 ^§^	2.5 ± 0.8	2.4 ± 0.7	14.8 ± 2.8 *^§^	20.1 ± 3.9 ^§^	12.2 ± 2.1 *	15.9 ± 4.0
3	3.1 ± 1.1 *^§^	2.6 ± 0.9 ^§^	2.1 ± 0.7	2.3 ± 0.7	14.3 ± 2.9 *^§^	19.8 ± 4.2 ^§^	11.5 ± 1.8 *	15.9 ± 4.5
4	2.6 ± 0.8 *^§^	2.2 ± 0.7 ^§^	2.0 ± 0.4	2.0 ± 0.4	10.0 ± 1.7 *^§^	13.9 ± 3.3 ^§^	8.5 ± 1.1 *	10.9 ± 2.1
5	2.3 ± 0.7 *^§^	2.0 ± 0.6	1.9 ± 0.4	1.8 ± 0.5	9.5 ± 1.6 *^§^	12.6 ± 2.9 ^§^	7.8 ± 1.1 *	9.5 ± 1.7
6	2.1 ± 0.8	2.0 ± 0.6 ^§^	1.9 ± 0.6	1.8 ± 0.5	8.9 ± 1.6 *^§^	11.6 ± 2.5 ^§^	7.5 ± 1.0 *	9.5 ± 1.7
ZW/ZZ	1.0 ± 0.0 *	2.3 ± 0.7	1.0 ± 0.0*	2.1 ± 0.5	13.1 ± 1.8 *^§^	15.7 ± 3.0 ^§^	10.2 ± 2.8 *	13.6 ± 3.3

* sex difference in the same species, Mann–Whitney test, *p* < 0.001; ^§^ species difference in the same sex, Mann–Whitney test, *p* < 0.001.

**Table 3:** Heterochiasmy and sexual dimorphism in birds.

Species	Genetic Map Length (cM)		References	Heterochiasmy Index	Sexual Dimorphism
	Female	Male			
Domestic goose (*Anser anser*) ^a^	3655	3030	[16]	0.19	low (weight) [30]
Turkey (*Meleagris gallopavo*) ^b^	2077	2431	[31]	−0.16	high (weight, plumage) [32]
Domestic chicken (*Gallus domesticus*) ^a^	3310	3285	[33]	0.01	high (weight, plumage) [34]
Domestic chicken (*Gallus domesticus*) ^b^	3098	3145	[35]	−0.02	high (weight, plumage) [34]
Japanese quail (*Coturnix japonica*) ^a^	2815	2815	[36]	0.00	high (weight, plumage) [37]
Pigeon (*Columba livia*) ^c^	3135	3235	[38]	−0.03	low (size) [39]
Siberian jay (*Perisoreus infaustus*) ^b^	998	774	[40]	0.25	none [41]
Pale martin (*Riparia diluta*) ^a^	2380	2450	this paper	−0.03	none [19]
Barn swallow (*Hirundo rustica*) ^a^	2815	2430	this paper	0.15	moderate (tail length) [42]
Great reed warbler (*Acrocephalus arundinaceus*) ^b^	858	552	[43]	0.43	none [44]
Blue tit (*Parus caeruleus*) ^b^	1046	887 ^b^	[45]	0.16	low (plumage) [46]
Collared flycatcher (*Ficedula albicollis*) ^b^	1627	1982	[47]	−0.20	low (plumage) [48]
Zebra finch (*Taeniopygia guttata*) ^a^	2335	2310	[36]	0.01	high (plumage) [49]

^a^—MLH1 mapping; ^b^—Linkage mapping; ^c^—Recombination nodule mapping (electron microscopy).

## Supplementary Materials

The following are available online at https://www.mdpi.com/2073-4425/11/10/1119/s1. Figure S1: Idiograms of pachytene karyotypes of barn swallow (a) and pale martin (b), Table S1: Number of specimens examined and the coordinates of the trapping localities, Table S2: Mann–Whitney test for sex difference in the same species and species difference in the same sex, Supplementary file 1: raw data.
