# Supplementary material for: Heterochiasmy and Sexual Dimorphism: The Case of the Barn Swallow (Hirundo rustica, Hirundinidae, Aves)
Source: Genes (Basel). 2020 Sep 24;11(10):1119. doi: 10.3390/genes11101119 (PMC7650650; doi:10.3390/genes11101119)
Supplement: Supplementary file 1 [file genes-11-01119-s001.zip › genes-938972-supplementary/genes-938972-suppl.docx]

**Table S1.** Number of specimens examined and the coordinates of the trapping localities

| Species | Sex | N specimens | Trapping locality | |
| --- | --- | --- | --- | --- |
|  |  |  | Latitude | Longitude |
| Barn Swallow | females | 3 | 54.81 | 83.09 |
| Barn Swallow | males | 5 | 54.87 | 83.14 |
| Pale Martin | females | 3 | 54.96 | 83.17 |
| Pale Martin | males | 3 | 54.96 | 83.17 |
| Pale Martin | males | 3 | 56.26 | 84. 56 |

**Table S2.** Mann-Whitney test for sex difference in the same species and species difference in the same sex

| Trait | Z | p-value | N Barn Swallow Females | N Barn Swallow Males |
| --- | --- | --- | --- | --- |
| SC1 length | -14.0 | 0.000000 | 182 | 275 |
| SC 1 MLH1 | 9.2 | 0.000000 | 182 | 275 |
| SC2 length | -14.3 | 0.000000 | 181 | 275 |
| SC 2 MLH1 | 6.9 | 0.000000 | 181 | 275 |
| SC3 length | -13.9 | 0.000000 | 181 | 275 |
| SC 3 MLH1 | 5.0 | 0.000001 | 181 | 275 |
| SC4 length | -14.7 | 0.000000 | 182 | 271 |
| SC 4 MLH1 | 6.3 | 0.000000 | 182 | 271 |
| SC5 length | -12.8 | 0.000000 | 177 | 272 |
| SC 5 MLH1 | 4.6 | 0.000003 | 177 | 272 |
| SC6 length | -13.5 | 0.000000 | 177 | 270 |
| SC 6 MLH1 | 0.8 | 0.407027 | 177 | 270 |
| SCZZ/ZW length | -13.6 | 0.000000 | 127 | 275 |
| SC ZZ/ZW MLH1 | -15.2 | 0.000000 | 127 | 275 |
| Total autosomal SC length | -10.7 | 0.000000 | 182 | 275 |
| Total autosomal MLH1 | 12.1 | 0.000000 | 182 | 275 |
|  |  |  |  |  |
| Trait | Z | p-value | N Pale Martin Females | N Pale Martin Males |
| SC1 length | -12.6 | 0.000000 | 145 | 291 |
| SC 1 MLH1 | -0.6 | 0.544888 | 145 | 291 |
| SC2 length | -12.7 | 0.000000 | 145 | 291 |
| SC 2 MLH1 | 0.3 | 0.741441 | 145 | 291 |
| SC3 length | -14.0 | 0.000000 | 145 | 275 |
| SC 3 MLH1 | -2.1 | 0.033333 | 145 | 275 |
| SC4 length | -13.9 | 0.000000 | 145 | 281 |
| SC 4 MLH1 | 1.0 | 0.310961 | 145 | 281 |
| SC5 length | -11.9 | 0.000000 | 143 | 256 |
| SC 5 MLH1 | 2.2 | 0.026773 | 142 | 256 |
| SC6 length | -14.0 | 0.000000 | 145 | 266 |
| SC 6 MLH1 | 0.7 | 0.486402 | 143 | 266 |
| SCZZ/ZW length | -9.6 | 0.000000 | 90 | 291 |
| SC ZZ/ZW MLH1 | -13.4 | 0.000000 | 90 | 291 |
| Total autosomal SC length | -14.0 | 0.000000 | 145 | 293 |
| Total autosomal MLH1 | -6.9 | 0.000000 | 145 | 293 |
|  |  |  |  |  |
| Trait | Z | p-value | N Barn Swallow Females | N Pale Martin Females |
| SC1 length | 9.7 | 0.000000 | 182 | 145 |
| SC 1 MLH1 | 10.4 | 0.000000 | 182 | 145 |
| SC2 length | 8.8 | 0.000000 | 181 | 145 |
| SC 2 MLH1 | 8.9 | 0.000000 | 181 | 145 |
| SC3 length | 9.8 | 0.000000 | 181 | 145 |
| SC 3 MLH1 | 8.1 | 0.000000 | 181 | 145 |
| SC4 length | 8.7 | 0.000000 | 182 | 145 |
| SC 4 MLH1 | 7.1 | 0.000000 | 182 | 145 |
| SC5 length | 9.9 | 0.000000 | 177 | 143 |
| SC 5 MLH1 | 5.1 | 0.000000 | 177 | 142 |
| SC6 length | 9.4 | 0.000000 | 177 | 145 |
| SC 6 MLH1 | 2.5 | 0.011300 | 177 | 143 |
| SCZZ/ZW length | 5.1 | 0.000000 | 127 | 90 |
| SC ZZ/ZW MLH1 | 0.0 | 1.000000 | 127 | 90 |
| Total autosomal SC length | 4.1 | 0.000039 | 182 | 145 |
| Total autosomal MLH1 | 12.7 | 0.000000 | 182 | 145 |
|  |  |  |  |  |
| Trait | Z | p-value | N Barn Swallow Males | N Pale Martin Males |
| SC1 length | 14.5 | 0.000000 | 275 | 291 |
| SC 1 MLH1 | 4.9 | 0.000001 | 275 | 291 |
| SC2 length | 14.5 | 0.000000 | 275 | 291 |
| SC 2 MLH1 | 5.5 | 0.000000 | 275 | 291 |
| SC3 length | 13.1 | 0.000000 | 275 | 275 |
| SC 3 MLH1 | 3.5 | 0.000537 | 275 | 275 |
| SC4 length | 14.1 | 0.000000 | 271 | 281 |
| SC 4 MLH1 | 2.8 | 0.005927 | 271 | 281 |
| SC5 length | 14.7 | 0.000000 | 272 | 256 |
| SC 5 MLH1 | 3.7 | 0.000198 | 272 | 256 |
| SC6 length | 13.0 | 0.000000 | 270 | 266 |
| SC 6 MLH1 | 3.5 | 0.000458 | 270 | 266 |
| SCZZ/ZW length | 11.0 | 0.000000 | 275 | 291 |
| SC ZZ/ZW MLH1 | 2.3 | 0.021828 | 275 | 291 |
| Total autosomal SC length | 2.6 | 0.008338 | 275 | 293 |
| Total autosomal MLH1 | -3.4 | 0.000584 | 275 | 293 |


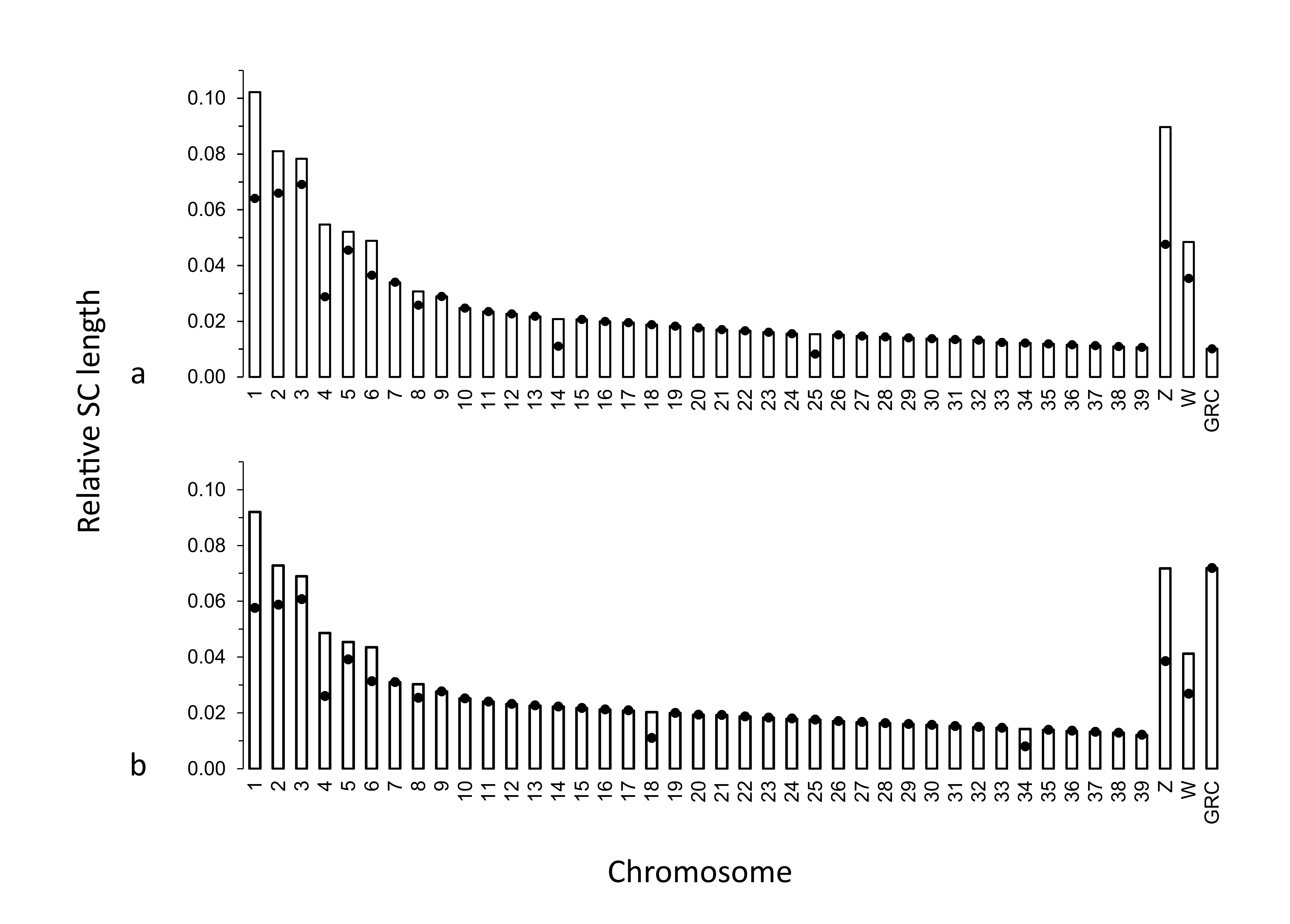


**Figure S1.** Idiograms of pachytene karyotypes of Barn Swallow (a) and Pale Martin (b). Y-axis shows relative length of the chromosome. Numbers indicate the autosomal SCs, letters – ZZ, ZW and GRC chromosomes.
